# Inhibitor binding mode and allosteric regulation of Na^**+**^-glucose symporters

**DOI:** 10.1038/s41467-018-07700-1

**Published:** 2018-12-07

**Authors:** Paola Bisignano, Chiara Ghezzi, Hyunil Jo, Nicholas F. Polizzi, Thorsten Althoff, Chakrapani Kalyanaraman, Rosmarie Friemann, Matthew P. Jacobson, Ernest M. Wright, Michael Grabe

**Affiliations:** 1 Department of Pharmaceutical Chemistry, University of California, San Francisco, CA 94158 USA; 2 Cardiovascular Research Institute, University of California, San Francisco, CA 94158 USA; 3 Department of Physiology, David Geffen School of Medicine at UCLA, Los Angeles, CA 90095-1751 USA; 4 Department of Chemistry and Molecular Biology, Centre for Antibiotic Resistance (CARe) at University of Gothenburg, SE-40530 Gothenburg, Sweden

## Abstract

Sodium-dependent glucose transporters (SGLTs) exploit sodium gradients to transport sugars across the plasma membrane. Due to their role in renal sugar reabsorption, SGLTs are targets for the treatment of type 2 diabetes. Current therapeutics are phlorizin derivatives that contain a sugar moiety bound to an aromatic aglycon tail. Here, we develop structural models of human SGLT1/2 in complex with inhibitors by combining computational and functional studies. Inhibitors bind with the sugar moiety in the sugar pocket and the aglycon tail in the extracellular vestibule. The binding poses corroborate mutagenesis studies and suggest a partial closure of the outer gate upon binding. The models also reveal a putative Na^+^ binding site in hSGLT1 whose disruption reduces the transport stoichiometry to the value observed in hSGLT2 and increases inhibition by aglycon tails. Our work demonstrates that subtype selectivity arises from Na^+^-regulated outer gate closure and a variable region in extracellular loop EL5.

## Abstract (web summary)

Sodium-dependent glucose transporters (SGLTs) transport sugars across the plasma membrane and play important roles in renal sugar reabsorption. Here authors develop structural models of human SGLT1/2 (hSGLT1/2) in complex with inhibitors which helps to understand inhibitor subtype selectivity.

## Introduction

Among the six human sodium-dependent glucose transporter (SGLT) subtypes widely expressed in the small intestine, kidney, lung, muscle, and brain^1^, hSGLT1 is the primary transporter in the intestine, while glucose reabsorption in the kidney is accomplished mostly by hSGLT2. Patients with mutations in the hSGLT2 gene develop a benign disorder called *familial renal glucosuria* (FRG), in which sugar reabsorption in the kidneys is impaired; however, they do not suffer any long-term consequences^2^. Thus, hSGLT2 inhibition has been a primary focus of type 2 diabetes (T2DM) research in the past decade^3^. Currently approved hSGLT2 inhibitors—glucosides containing a sugar moiety connected to an aromatic tail referred to as an aglycon—were also shown to reduce heart failure hospitalization rates by 35% compared to other diabetes treatments while also cutting deaths from any cause by 32%^4^. While very promising, these drugs are not free from side effects^5^, and the lack of knowledge concerning the molecular determinants of action poses a barrier to developing new chemotypes with an improved therapeutic window. Structurally, SGLTs fall into the large leucine-transporter (LeuT) family^6^, and they work by means of an alternating access mechanism in which they first bind Na^+^ and sugar from the extracellular side in a so-called outward-facing conformation and then transition to an inward-facing conformation to release their cargo to the cytoplasm. The structural basis of how inhibitors block transport is not known.

While recent studies have attempted to dock phlorizin-derived compounds into inward-facing models of hSGLTs^7^, the Wright lab has demonstrated that SGLT2 inhibitors bind from the extracellular solution likely stabilizing a Na^+^-bound, outward-facing conformation^8^. Thus, a detailed molecular view of this interaction is not possible without a reliable outward-facing model of SGLTs. While the closely related bacterial homolog from *Vibrio parahaemolyticus* (vSGLT) has been solved in the apo-^9^ and sugar-bound^10^ conformations, both structures are inward facing, and although suitable for modeling hSGLT–glucose complexes, our attempts to dock phlorizin into hSGLT models built upon these templates failed. Previously, members of the superfamily have been solved in the outward-facing state^11–13^, but the sequence identities are far too low to make reliable SGLT homology models (<8%). The outward-facing structure of the *N*-acetylneuraminic acid transporter from *Proteus mirabilis* (SiaT) was determined at 1.95 Å resolution^14^, and this LeuT-fold transporter shares moderate sequence identity with SGLTs (~24% identity/~46% similarity), a value that is significantly higher than any other LeuT-fold transporter of known structure except for vSGLT, which has similar sequence identity to the human SGLTs as SiaT. Here we show with a combination of computational and experimental approaches that this outward-facing SiaT structure serves as a good template for understanding inhibitor binding and the subtype specificity of human SGLTs.

## Results

### The hSGLT1–phlorizin complex explains mutagenesis data

Our initial attempts to dock small molecules into inward-facing models of hSGLT built on the available vSGLT structures provided mixed results. Glucose adopts a binding pose similar to the one observed for galactose in vSGLT. However, phlorizin fails to adopt a reasonable pose as the glucose moiety does not occupy the sugar binding site nor does the molecule contact any of the protein residues known to influence inhibition (Supplementary Methods and Supplementary Fig. 1). Therefore, we turned to SiaT as a potential outward-facing template. We first used a combination of sequence^15^ and structure-based^16^ methods to reach a consensus alignment between the SGLTs and SiaT (Supplementary Methods and Supplementary Fig. 2), and we then used the alignment to create outward-facing SGLT models based on the SiaT structure with Modeller^17^. Initial glucose docking studies were carried out on these models to confirm the role of known residues in the glucose binding site based on published mutagenesis data and interactions observed in the inward-facing vSGLT co-crystal (see Methods).

**Fig. 1:**
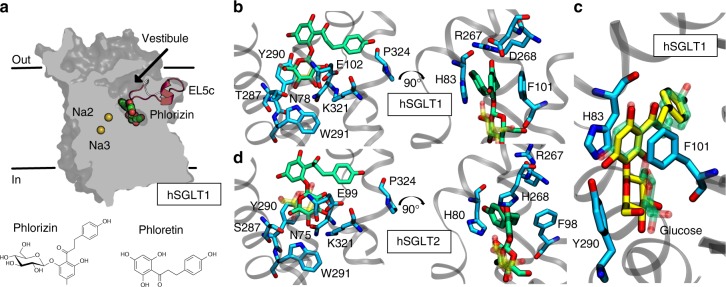
Predicted binding mode of phlorizin to hSGLT1 and hSGLT2. **a** Phlorizin bound to the outward-facing hSGLT1. The protein is gray, phlorizin is green and red, and Na^+^ at the putative Na2/Na3 sites are yellow. EL5c (red) is represented as cartoon. The 2D structures of phlorizin and phloretin are also shown. **b** Close-up view of phlorizin bound to hSGLT1. The sugar moiety occupies the sugar binding site aligning with the docked glucose (transparent yellow/red) where it makes excellent hydrogen bonding with the protein. The aglycon tail creates face-to-face aromatic π–π interactions with H83 and F101 and a cation–π interaction with R267 on EL5_C_. **c** Phloretin and glucose bound to hSGLT1. Phloretin (yellow/red) superposes over the aglycon tail from the phlorizin molecule (transparent green/red) in **b**, while glucose (yellow/red) aligns well with the sugar moiety. **d** Close-up view of phlorizin bound to hSGLT2. The binding mode is conserved in hSGLT2, although the aglycon tail is more tightly packed due to an aromatic cage that forms around the central ring made up of residues H80, F98, and H268 on EL5_C_

Next, we docked phlorizin into the outward-facing model of hSGLT1 using a flexible docking procedure, which allows for motion in the small molecule around its rotatable bonds while keeping the protein fixed. We then rescored each of the top 10 binding poses, allowing the protein side chains within 4 Å of phlorizin to relax with the MM/GBSA^18^ protocol. The top-ranked pose (green/red) occupies the same vestibule found for the MTS-TAMRA (2-((5(6)-tetramethyl-rhodamine)carboxylamino)ethyl methanethiosulfonate) reagent^19^ and shows overlap of the phlorizin sugar moiety with the redocked glucose (transparent yellow/red), while the aglycon tail occupies the outer vestibule pointing towards the extracellular space (Fig. 1a, b). The sugar moiety makes hydrogen bond interactions with residues N78, H83, E102, Y290, W291, and K321, which have all been shown to affect both phlorizin-dependent inhibition and sugar transport^20^. Y290 has stacking interactions with both glucose and phlorizin, but its hydroxyl group also hydrogen bonds directly to the hydroxyl at the C1 position of phlorizin. Meanwhile, Y290’s ability to hydrogen bond to transmembrane segment 1 (TM1) is thought to play a role in sugar transport in vSGLT^9^. We tested the importance of hydrogen bonding by mutating Y290 to F in hSGLT1 and measuring the glucose transport and the inhibition of steady-state, glucose-induced Na^+^ current as a function of external inhibitor concentration. The inhibitory constant (*K*_i_) for phlorizin to the wild-type transporter was 0.22 ± 0.04 μM under these conditions, and the value increased eightfold in the Y290F mutant (1.8 ± 0.4 μM) consistent with the loss of a single hydrogen bond to the aglycon tail. Glucose transport exhibited a decrease in glucose apparent affinity (*K*_0.5_) from 0.9 ± 0.1 mM for wild type to 35 ± 10 mM^21^—a nearly 35-fold change, thus confirming the importance of the chemistry at this site for both molecules. Glucose transport may be more impacted due to its smaller size and the polar nature of the interactions with the transporter, while the mostly hydrophobic nature of the interactions between the aglycon tail and the transporter are not impacted by the Y290F mutation. Meanwhile, F101 in TM2 exhibits π–π stacking with aromatic elements in the aglycon tail, but no interactions with the sugar moiety (Fig. 1b) corroborating the experimental finding that F101C increases the phlorizin *K*_i_ 170-fold while having no effect on sugar transport^20^. To assess the robustness of the predicted pose, we docked the aglycon tail alone, which is called phloretin, into hSGLT1. The smaller phloretin molecule (yellow/red) perfectly overlaps with the aglycon tail of phlorizin providing additional confidence in the pose (Fig. 1c).

### Structural determinants of inhibitor subtype selectivity

Next, we wanted to explore the differences in inhibitor binding between hSGLT1 and hSGLT2 in an attempt to understand why hSGLT2 is more easily inhibited by phlorizin-derived molecules. We started by docking phlorizin into an outward-facing model of hSGLT2 constructed as described for hSGLT1. The inhibitor-binding mode is conserved between both transporters (Fig. 1d). The sugar moiety recapitulates the same interaction landscape observed for hSGLT1, which was expected since the glucose binding sites are conserved. In addition, many aromatic interactions with the aglycon tail are preserved (H80/H83 and F98/F101, hSGLT2/hSGLT1 numbering, respectively); however, a notable sequence difference exists between both transporters in the C-terminal end of the long extracellular loop 5 (EL5_C_) that connects TM5 to TM6 (Supplementary Fig. 2). hSGLT2 has a third aromatic residue (H268) that stacks in a parallel displacement fashion with F98 to create an aromatic cage (H80, F98, H268) around the central ring of the aglycon tail. hSGLT1 does not form this cage because 268 is an aspartic acid, and D268 creates a salt bridge with the conserved R267 redirecting the guanidinium group towards a cation–π interaction with the inhibitor. The introduction of the histidine in position 268 in hSGLT2 confers additional packing interactions absent in hSGLT1.

**Fig. 2:**
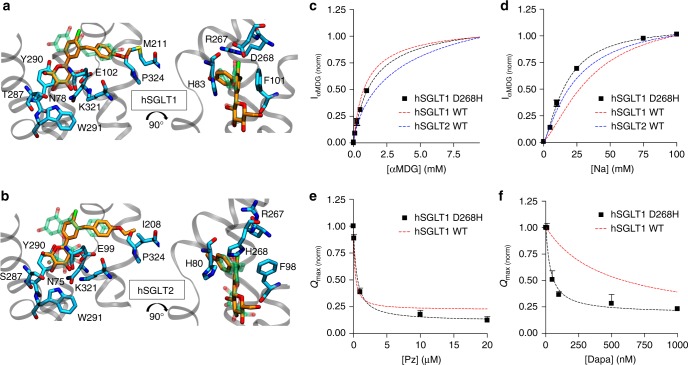
Predicted binding mode of dapagliflozin to hSGLT1 and hSGLT2. Dapagliflozin (orange/red) adopts a similar pose to phlorizin (transparent green/red) in both hSGLT1 (**a**) and hSGLT2 (**b**). Interactions in the sugar binding site and extracellular vestibule are preserved including the electrostatic interaction with R267 (hSGLT1) and the aromatic cage formed by H80, F98, and H268 (hSGLT2). **c** αMDG hSGLT1 D268H (black squares) sugar currents. αMDG currents were measured at *V*_m_ = −50mV. The red dashed curve is a representative, wild-type hSGLT1 response. For the D268H mutant *K*_0.5_ = 1.2 ± 0.1 mM, while wild-type hSGLT1 *K*_0.5_ = 0.9 ± 0.1 mM. For **c** and **d**, data are normalized to the current measured at 10 mM αMDG. **d** Na^+^ dependence of αMDG currents measured in 10 mM αMDG at *V*_m_ = −50mV. For hSGLT1 D268H *K*_0.5_ = 17 ± 1.2 mM and for wild-type hSGLT1 *K*_0.5_ = 36 ± 1 mM. Hill coefficients hSGLT1 D268H and wild-type were 1.62 ± 0.03 and 1.61 ± 0.1. **e** Phlorizin and **f** dapagliflozin effect on the *Q*_max_ for hSGLT1 D268H, we estimated a *K*_i_ = 0.3 ± 0.1 and 0.035 ± 0.011 µM for phlorizin and dapagliflozin, respectively, while the wild-type *K*_i_ = 0.22 ± 0.04 and 0.45 ± 0.02 µM. For **c**–**f**, each data point is the mean ± SEM of *n* ≥ 5 oocytes

Analysis of the phlorizin-docked complexes revealed only subtle structural differences in the binding poses, which we hesitate to attribute to the increased inhibition of hSGLT2 over hSGLT1 without further analysis since these are homology models and phlorizin has rather low subtype selectivity (eightfold increase in inhibition of hSGLT2 over hSGLT1). To gain greater insight into subtype specificity, we next docked the T2DM drug dapagliflozin into both outward-facing models to identify structural differences between the proteins that may explain why this drug has a >100-fold higher potency for hSGLT2^22^. Dapagliflozin docks into both transporters in a very similar manner with the sugar moiety occupying the glucose binding site and the aglycon tail occupying the extracellular vestibule (Fig. 2). Additionally, overlap with the phlorizin structures (transparent) reveals that the two molecules engage with the transporters similarly. The aromatic cage around the aglycon tail persists in hSGLT2, and again R267 has a cation–π interaction with the tail in hSGLT1. Again, as for phlorizin, the introduction of the histidine in position 268 in hSGLT2 confers additional packing interactions absent in hSGLT1. This difference might provide some degree of selectivity, and we tested this possibility experimentally.

To do so, we focused on two mutants: hSGLT1 D268H, which mimics EL5_C_ of hSGLT2, and hSGLT1 R267C, which although conserved, only interacts with phlorizin in our hSGLT1-docked model, but not in hSGLT2 (Fig. 2a, b). We expressed both mutants in *Xenopus laevis* oocytes and recorded sugar-induced currents (Fig. 2 and Table 1) elicited by α-methyl-d-glucopyranoside (αMDG)—a glucose analog, which we interchangeably refer to as glucose throughout. Unfortunately, the R267C mutant was not expressed in oocytes, but D268H exhibited an apparent Na^+^ affinity (17 ± 1.2 mM) and apparent *K*_0.5_ for glucose (1.2 ± 0.1 mM) both similar to wild type (36 ± 1 and 0.9 ± 0.1 mM, respectively). Moreover, hSGLT1 D268H is inhibited by phlorizin to the same degree as wild type (*K*_i_ = 0.3 ± 0.1 and 0.22 ± 0.04 μM, respectively). However, the *K*_i_ for dapagliflozin is 13-fold lower (0.035 ± 0.011 μM) than the wild-type *K*_i_ (0.45 ± 0.02 μM), indicating that D268H in EL5_C_ contributes to the selectivity of dapagliflozin but not phlorizin. The docked structures may rationalize this difference. Phlorizin possesses a central aromatic ring which protrudes towards EL5_C_ making either a cation–π interaction with R267 in hSGLT1 (Fig. 1b) or π–π and Van der Waals packing with the imidazole of H268 in hSGLT2 (Fig. 1d). Meanwhile, dapagliflozin lies deeper in the binding pocket, and its central ring is too far to make a strong cation–π interaction with R267 in hSGLT1 (Fig. 2a), but it is still able to establish excellent interactions with H268 in hSGLT2 (Fig. 2b). Thus, the introduction of D268H in hSGLT1 may impact dapagliflozin binding more than phlorizin due to deeper binding of dapagliflozin.

**Table 1:** Measured *K*_i_ and *K*_0.5_ values for hSGLT2, hSGLT1 and hSGLT1 mutants

Transporter	*K*_0.5_ αMDG (mM)	*K*_0.5_ Na^+^ (mM)	*K*_i_ phlorizin (μM)	*K*_i_ phloretin (μM)	*K*_i_ dapagliflozin (μM)	*K*_i_ dapa-aglycon (μM)
hSGLT1						
Wild-type	0.9 ± 0.1	36 ± 1	0.22 ± 0.04	55 ± 12	0.45 ± 0.02	425 ± 50
F101C	1.1 ± 0.2^20^	9 ± 1^20^	37 ± 12^20^			
D268H	1.2 ± 0.1	17 ± 1.2	0.3 ± 0.1		0.035 ± 0.011	
T287C	1.4 ± 0.2^19^	6 ± 0.2^19^	0.2 ± 0.1			
Y290C	>100^20^	46 ± 5^20^	36 ± 6^20^			
Y290F	35 ± 10^21^	20 ± 1^21^	1.8 ± 0.4			
S392A/C	>100^20^	122 ± 12^20^	4 ± 1^20^			
S393A/C	4 ± 1^20^	32 ± 3^20^	0.5 ± 0.2^20^			
T395A	34 ± 4	104 ± 50	0.35 ± 0.10	20 ± 7	0.4 ± 0.1	187 ± 80
Q457C	13 ± 2^20^	34 ± 2^20^	6 ± 1^20^			
F453C	1.8 ± 0.2^20^	21 ± 1^20^	1 ± 0.3^20^			
hSGLT2						
Wild-type	4.4 ± 0.1	22 ± 1	0.03 ± 0.01	27 ± 3	0.004 ± 0.001	110 ± 30

All parameters were estimated from SGLT current measurement in oocytes at 22 °C and each parameter is given as the mean and ±SEM of three to five estimates. The results for wild-type hSGLT1 and hSGLT2 agree with previous reports^20, 27, 65^. Superscripts refer to data obtained for hSGLT1 mutants expressed in oocytes and reported previously^19–21^. Data reported without superscripts were generated for this study

### The outer gate partially closes over bound inhibitors

**Fig. 3:**
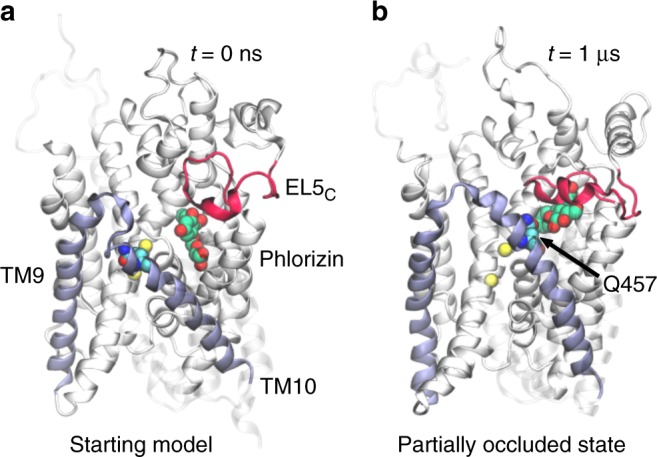
Outer gate closure explains the impact of TM9-10 residues on substrate binding. **a** The outer gate residue Q457 on TM10 does not interact with phlorizin (green/red) in the outward-facing model of hSGLT1. **b** A molecular dynamics simulation of the hSGLT1–phlorizin complex reveals significant movement in the TM9-10 outer gate bringing Q457 into direct contact with phlorizin. Additionally, EL5_C_ (red) closes over the top of the inhibitor. This figure is a snapshot from the trajectory at 1 μs. Lipids and water are not shown for clarity

Our analysis has focused on residues in direct contact with the inhibitors, and now we turn our attention to mutations in hSGLT1 that impact glucose and phlorizin binding, but do not establish direct contact based on the model. The first two positions are F453 and Q457, which are on the same face of helix TM10 in the extracellular vestibule, are both solvent exposed, and more than 10 Å away from phlorizin (Fig. 3a). Nonetheless, mutations to cysteine at these positions increase the *K*_0.5_ of glucose 2- and 14-fold^20^, respectively, while they increase the *K*_i_ of phlorizin inhibition 4.5- and 27-fold^20^, respectively. We believe that their influence can be understood through an induced-fit hypothesis since the extracellular half of TM9 and TM10 and the small intervening loop form an outer gate in the inward-facing states of vSGLT^10^, Mhp1^23^, BetP^24^, and LeuT^13^ that closes over the substrate to occlude it from the extracellular solution. If TM9-10 is dynamic and phlorizin binding results in partial closure of the gate, it may bring the extracellular end of TM10 into contact with the substrates (sugar and inhibitors) as in the inward-facing hSGLT1–glucose modeled complex and the inward-facing occluded vSGLT structure^10^ (Supplementary Figs. 1a and 3).

To test our induced-fit hypothesis, we carried out 1 μs of fully atomistic molecular dynamics (MD) simulations on the hSGLT1–phlorizin bound complex to determine how elements of the outer gate behave. During the simulation (Supplementary Movie 1), we observe substantial movement in the TM9-10 outer gate (ice blue) bringing Q457 into close proximity with the inhibitor while at the same time heading toward an occluded state of the transporter in which both the outer gate and inner gate are closed (Fig. 3). Partial gate closure occurs via a rigid rotation of TM9 and a bending of TM10 around a central kink, but EL5_C_ (red) also plays a role in closure by forming a cap over the aglycon tail of the inhibitor in a lid-like fashion (Fig. 3b).

### Disruption of the Na3 site enhances aglycon potency

**Fig. 4:**
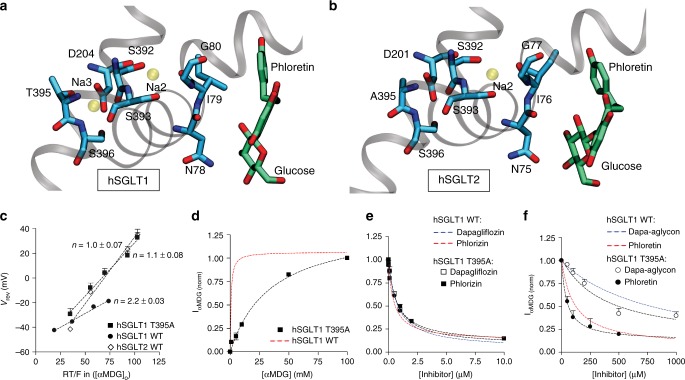
Allosteric communication between sodium and substrate sites. **a** The hSGLT1–phlorizin model shows the location of Na^+^ (transparent yellow) in the Na2 site over 8 Å from the inhibitor and the putative Na3 site 14 Å away. The Na2 Na^+^ interacts with the side chains of S392 and S393 and the carbonyl backbone of I79, while the ion at Na3 is stabilized by backbone carbonyls, side chains of T395 and S396, and bidentate interaction with D204. **b** The hSGLT2–phlorizin model shows the location of the conserved Na2 binding site (transparent yellow), but the putative Na3 site is lost by the hydrophobic substitution A395. **c** hSGLT1 T395A and wild-type hSGLT1 and hSGLT2 stoichiometries were determined from the reversal potential (*V*_rev_). The inverse of the slope, Na^+^-to-glucose coupling ratio, *n*, is 1-to-1 Na^+^-to-substrate stoichiometry for the hSGLT2 wild-type and the T395A hSGLT1 mutant and 2-to-1 for wild-type hSGLT1. **d** αMDG dependence of hSGLT1 T395A (black squares) sugar current in injected oocytes. *K*_0.5_ = 34 ± 4 mM compared to *K*_0.5_ = 0.9 ± 0.1 mM for wild type. Data in **d**–**f** are normalized to the current measured in 10 mM αMDG without inhibitors, and the red dashed curves are representative, wild-type hSGLT1 responses. **e** Phlorizin (black squares) and dapagliflozin (open squares) effect on αMDG currents for hSGLT1 T395A in 30 mM αMDG. For hSGLT1 T395A *K*_i_ = 0.35 ± 0.10 and 0.4 ± 0.1 µM for phlorizin and dapagliflozin, respectively, while wild type estimated *K*_i_ = 0.22 ± 0.04 and 0.45 ± 0.02 µM for phlorizin and dapagliflozin, respectively. **f** Phloretin (black circles) and dapa-aglycon (open circles) effect on αMDG currents for hSGLT1 T395A in 30 mM αMDG. For hSGLT1 T395A *K*_i_ = 20 ± 7 and 187 ± 80 µM for phloretin and dapa-aglycon, respectively, while the wild-type *K*_i_ = 55 ± 12 and 425 ± 50 µM for phloretin and dapa-aglycon, respectively. Each data point is the mean ± SEM of *n* = 10 oocytes (**c**), *n* ≥ 5 oocytes (**d**), and *n* ≥ 7 oocytes (**e**, **f**)

Similar to sites in the extracellular gate, the mutation S392A impacts glucose transport and phlorizin potency^20^, but it does not directly contact the molecules. Unlike the outer gate positions, S392 fails to make direct contact in both the inward-facing and outward-facing models, and it is located more than 10 Å away from the substrate in the outward-facing model (Fig. 4a). It forms part of the Na2 Na^+^ binding site based on the vSGLT structures^9, 10^, and it is conserved throughout the solute sodium symporter family (see Supplementary Fig. 2 for partial list). Thus, we hypothesize that S392A disrupts Na^+^ binding and subsequently reduces substrate binding through allosteric means. Experimentally, it is known that Na^+^ binding precedes substrate binding from the extracellular face^25, 26^, as ion binding opens access to the substrate and inhibitor binding sites^19^ while also pre-organizing the sites for increased binding affinity. Our recent simulations of SiaT support the claim that ions aid in pre-organizing residues in the substrate binding site, as we showed that the bound sialic acid was far more stable in the binding site when Na^+^ was bound at Na2 and/or Na3, despite no direct contact between the substrate and ions^14^. A network of interactions exists in all LeuT transporters extending from the Na2 site to the unwound section of TM1 that directly contacts the transported substrate (Fig. 4a, b), and when Na^+^ binding at Na2 is impaired, we hypothesize that TM1 does not adopt a conformation compatible with tight sugar and inhibitor binding.

Motivated by the impact that Na^+^ binding to the Na2 site has on substrate and inhibitor binding, coupled with the fact that EL5_C_ explains only part of the selectivity towards hSGLT2, we decided to explore the primary difference between hSGLT1 and hSGLT2—the latter binds only a single Na^+^, while hSGLT1 binds 2 Na^+^. There is little information regarding the structural influence that binding 1 vs. 2 Na^+^ imparts to SGLTs; however, our experiments suggest that hSGLT1 binds glucose five times more tightly than hSGLT2 (Table 1), which may or may not be related to differences in stoichiometry^27^. Structural insight into ion binding comes from spectroscopic studies on LeuT^13^, which has a 2-to-1 stoichiometry, and Mhp1^28^ and vSGLT^29^, which bind a single Na^+^ at the Na2 site like hSGLT2. Interestingly, under saturating Na^+^ levels, presumably resulting in the binding of 2 Na^+^ ions, LeuT adopts an outward-open conformation with the TM9-10 gate wide open. However, saturating Na^+^ does not result in an outward-open conformation for Mhp1^28^ and vSGLT^29^. Thus, we hypothesize that the second Na^+^ binding event stabilizes the outer gate in an open conformation, while a single Na^+^ binding at the Na2 site does not efficiently hold the outer gate open. Since our model and simulation suggest an induced-fit mechanism of binding, the corollary of our hypothesis is that hSGLT2 may bind phlorizin-like molecules more tightly because the gate closes more easily over the inhibitors to make several contacts with residues belonging to the extracellular portion of TM10. To test this hypothesis, we attempted to abolish binding of Na^+^ to the additional Na site in hSGLT1 and measure the influence on inhibitors and substrate.

Unfortunately, the crystallographic position of the Na1 site in hSGLT1 is unknown. The LeuT X-ray structures revealed that a non-conserved Na^+^ binding site (termed Na1) directly coordinates the substrate and the protein^30^. Initial functional work on hSGLT1 seemed to suggest that Na^+^ also binds to a Na1-like site^31^ (termed Na1), but our attempts to simulate Na^+^ at this position in either single or double occupancy (at Na1 and Na2 sites, Supplementary Table 1) failed to provide a stable configuration (Supplementary Fig. 4a, b). Next, we searched for potential Na^+^ binding sites in the vicinity, and we identified a region in hSGLT1 that provides excellent Na^+^ coordination from two well-conserved polar groups (S396 and T395), an acidic residue (D204), and coordinating backbone carbonyl from S392, which also forms part of the Na2 site (Fig. 4a). This site is the proposed second Na^+^ binding site (termed Na3) which was resolved in the SiaT X-ray structure^14^. When we simulated hSGLT1 with Na^+^ in Na3, in single and double (Na2 and Na3) occupancy, the Na3 ion remained stable (Supplementary Fig. 4c, d). As one might expect, Na3 is present in hSGLT1, but not hSGLT2 or vSGLT (Supplementary Fig. 2). The critical difference with hSGLT2 is the hydrophobic substitution at T395 by alanine (Fig. 4).

To further assess the likelihood that Na3 is a suitable Na^+^ site, we carried out a comprehensive analysis of all known Na^+^ binding sites in the RCSB database^32^. While such analysis has been performed previously^33^, the dataset analyzed here is twice as large. Sodium sites have a five-point coordination arising from interactions with both side chain elements and main chain carbonyls. The putative Na3 site in hSGLT1 (Fig. 4a and Supplementary Fig. 5) is coordinated by Asp, Ser, and Thr side chains, which are the first, fourth, and fifth most common coordinating residues, as well as a Ser main chain interaction, which is the fourth most common main chain motif (Supplementary Fig. 6). Structurally similar coordination sites involving either a Ser/Thr at positions *i* and *i* + 1 along the primary sequence, as observed for both hSGLT1 and SiaT, returned the structurally unrelated sodium-calcium exchanger, which nonetheless binds Na^+^ with a similar coordination and also contains an acidic group. Lastly, D204 interacts with the Na^+^ in a bidentate fashion, which represents nearly half of all Asp residues in Na^+^ binding sites, several of which are pictured in Supplementary Fig. 5d. Thus, the Na3 site in hSGLT1 has all of the hallmarks of an excellent Na^+^ coordination site.

To determine if this putative position is a true sodium binding site, we expressed the hSGLT1 T395A mutant in *Xenopus* oocytes and recorded sugar-induced Na^+^ currents. The mutant displays markedly decreased apparent affinity for Na^+^ (104 ± 50 mM) compared to wild type (36 ± 1 mM), a loss of pre-steady-state currents that looks more hSGLT2-like than hSGLT1-like, and a decreased Na^+^-to-glucose coupling ratio from 2.2 ± 0.03 to 1.1 ± 0.08 as measured via reversal potentials (Fig. 4c), and confirmed by estimates from Na^+^ Hill coefficients that were reduced from 1.7 ± 0.03 to 1.1 ± 0.3 (Supplementary Table 2). Thus, experiments suggest that the Na3 site is the additional Na^+^ binding site, and T395A has hSGLT2-like properties with an apparent 1-to-1 stoichiometry. Next, we measured the apparent affinity for glucose to hSGLT1 T395A in non-saturating 100 mM external Na^+^ and found a 38-fold decrease from 0.9 ± 0.1 mM in wild type to 34 ± 4 mM (Table 1). This mutation has a dramatic effect on glucose affinity that is comparable to Na2 binding site mutations (S392A/C >100 mM and S393A/C ~4 mM)^20^. Interestingly, the mutant sugar *K*_0.5_ is eightfold lower than the value for wild-type hSGLT2, despite position 395 being an alanine in both proteins.

Next, we measured the inhibition constant of phlorizin to hSGLT1 T395A, and rather than increasing the inhibition strength, as we hypothesized, it decreased inhibition ~1.5-fold compared to the wild-type transporter (350 ± 100 vs. 220 nM, respectively). We believe that the decrease in affinity for sugar and the increase of *K*_i_ for phlorizin is explained by the lack of full Na^+^ occupancy at the Na2 site under these conditions, which results in failure to pre-organize the sugar binding site and suboptimal binding of glucose and the glucose moiety. We therefore tested the potency of the aglycon tail against this mutant. As we predicted, phloretin potency is increased 2.7-fold in the Na3-defective mutant (*K*_i_ = 20 ± 7 μM compared to 55 ± 12 μM for the wild type), which is marginally better than phloretin inhibition of hSGLT2 (*K*_i_ = 27 ± 3 μM). We also tested dapagliflozin inhibition of the hSGLT1 T395A mutant and determined that it was unchanged from wild type, while the dapa-aglycon, the aglycon tail of dapagliflozin, inhibited the mutant three times better (*K*_i_ = 187 ± 80 and 425 ± 50 μM, respectively). Finally, we attempted to introduce the Na3 site into hSGLT2 by mutating A395 to Thr (Supplementary Table 2). Unfortunately, this construct exhibited poor expression (about 10% of wild type), and it was not possible to determine the stoichiometry. Nonetheless, the mutant appears to have similar sugar and sodium affinity (4 ± 0.6 and 30 ± 10 mM, respectively) to wild type (4.4 ± 0.1 and 22 ± 1 mM, respectively), while inhibition by phloretin, dapa-aglycon, and phlorizin (*K*_i_ = 129 ± 70, >600, and 0.04 ± 0.01 μM, respectively) are all decreased when compared to wild type (*K*_i_ = 27 ± 3, 110 ± 30, and 0.03 ± 0.01 μM, respectively). The reduced inhibition by phlorizin-like molecules is what we expect if Na^+^ at the Na3 site stabilizes the transporter in an outward-facing conformation. Due to large standard error, we cannot comment on inhibition by dapagliflozin.

## Discussion

In this study, our goal was to provide a structural basis for the inhibition of SGLTs by drugs approved by the Food and Drug Administration for the treatment of T2DM. A hallmark of these drugs is their high potency and specificity for the hSGLT2 isoform uniquely expressed in the kidney, where they lower blood glucose levels by reducing glucose reabsorption. Our approach was to carefully construct structural models of hSGLT1 and hSGLT2 based on the crystal structures of two bacterial homologs, vSGLT and SiaT, dock the SGLT inhibitors into the homology models, and compare the docking poses with functional studies on SGLT mutant transporters. Our results reveal that the inhibitors bind to the Na^+^-bound, outward-facing conformation of hSGLT1 and hSGLT2 with the glucose moiety of the inhibitor in the sugar binding site and the aglycon tail in the external vestibule. We predict that subtype selectivity arises from two sources: (1) differences in the C-terminal end of EL5, and (2) allosteric control of the outer gate by Na^+^ binding.

The validity of the hSGLT1 and hSGLT2 structural models was demonstrated by: (1) the conservation of the hSGLT1–glucose interaction landscape in the inward- and the outward-facing states with respect to the sugar-bound vSGLT structure (Supplementary Figs. 1a and 3), and (2) phlorizin and dapagliflozin binding to the external surface—but not the cytoplasmic surface—consistent with functional studies^8^ and data generated here (Figs. 1 and 2 and Supplementary Fig. 1b and Table 1). The inhibitor binding site is remarkably similar for hSGLT1 and hSGLT2 in that the glucose moiety of the inhibitor, phlorizin and dapagliflozin, overlaps the glucose binding site (Figs. 1 and 2). This is not surprising since the coordinating residues are conserved between the two transporters and mutation of these residues disrupts sugar transport and inhibition by phlorizin (Table 1). Second, the aglycon tails, phloretin and dapa-aglycon, fit into a large vestibule leading from the external solution to the glucose binding site (Figs. 1 and 2, and ref. ^19^). Aglycon/vestibule interactions are dominated by π–π interactions and hydrophobic contact with F101 and H83 in hSGLT1 and F98 and H80 in hSGLT2. Mutation of F101 in hSGLT1 results in a 170-fold decrease in phlorizin inhibition with no change in glucose transport (Table 1). A significant difference between hSGLT2 and hSGLT1 that may contribute to its greater inhibition by phlorizin (eightfold) and dapagliflozin (>100-fold) is the presence of H268 in EL5_c_, which gives rise to an aromatic cage around the central ring of the aglycon composed of three residues rather than two (Fig. 1b, d). D268 is the equivalent residue in hSGLT1, and it forms a salt bridge with the adjacent conserved residue R267. Simply mutating D268 to histidine in hSGLT1, so that it resembles EL5_C_ in hSGLT2, leads to a 13-fold increase in dapagliflozin potency arising from improved packing interactions based on our docked models (Table 1). However, this single point mutation in EL5_C_ cannot explain the >100-fold selectivity of dapagliflozin for hSGLT2 over hSGLT1.

**Fig. 5:**
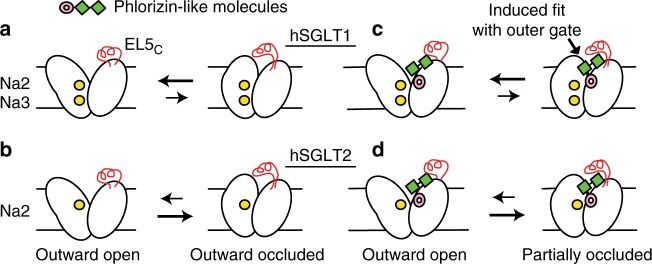
Allosteric model of inhibitor binding to SGLTs. The binding of phlorizin-like inhibitors to the outward-facing state of SGLTs leads to a partial closure of the TM9-10 outer gate in an induced fit mechanism. **a**, **c** Binding of 2 Na^+^ to hSGLT1 stabilizes the outer gate in an open conformation making it less favorable for the gate to close over the aglycon tails of inhibitors. **b**, **d** The absence of the Na3 site in hSGLT2 makes partial closure and inhibitor binding more favorable and therefore the inhibitors more potent. Additionally, elements of EL5_C_ (red) contribute to better binding to hSGLT2

One discrepancy between the structural models and functional data concerns the mutation of two residues on the solvent-exposed face of helix TM10, Q457, and F453 in hSGLT1, that decrease glucose transport and phlorizin-dependent inhibition despite no interactions in the models (Table 1). Our fully atomistic molecular dynamic simulations reveal substantial rotation of TM9 and bending in the external half of TM10 upon phlorizin binding bringing Q457 close to the inhibitor, while EL5_C_ forms a cap over the inhibitor (see Figs. 3 and 5 and Supplementary Movie 1). Thus, our simulations together with mutagenesis studies suggest that the inhibited state of the transporters involves rearrangements in the extracellular gate.

Another major difference between hSGLT2 and hSGLT1 is that glucose transport is driven by one Na^+^ ion, not two, and so we turned to Na^+^ stoichiometry as another potential determinant of inhibitor specificity. We identified a putative Na^+^ binding site in hSGLT1 (Na3), not present in hSGLT2 (Fig. 4), which is below the conserved Na2 site (see Supplementary Fig. 5 and ref. ^14^). The Na3 site is also present in SiaT^14^, but is distinct from the LeuT Na1 and BetP Na1’ sites, and while having the same name, it is not the same site identified as Na3 in GlyT2 (see Supplementary Fig. 7 for an overview of LeuT-fold Na^+^ binding sites). Mutation of a Na3 coordinating residue, T365A, in hSGLT1 dramatically reduces Na^+^ affinity, and impacts glucose transport and inhibitor potency. Most importantly, the T365A mutant is more easily inhibited by the aglycon tail of phlorizin and dapagliflozin (Table 1), supporting our hypothesis that inhibitors bind via an induced-fit mechanism as shown in the cartoon in Fig. 5. In this model, sites in EL5_C_ (red) provide some of the energetics of inhibitor binding, while also providing some of the subtype selectivity. A synergistic factor comes from partial closure of the outer gate, which is more energetically favorable with only a single Na^+^ at the Na2 site (hSGLT2) than it is with 2 Na^+^ ions bound (hSGLT1).

There are several lines of evidence supporting the hypothesis that Na^+^ allosterically controls the outer gate. Extensive biochemical data show that external Na^+^ is required for the alkylation of cysteine residues in the glucose binding site in hSGLT1^20^, and active site voltage clamp demonstrates that external Na^+^ controls the opening and closing of the external gate in real time (1–10 ms)^19^. In addition, mutation of Na2 site residues blocks the voltage-dependent opening and closing of the outer gates^31^. As already discussed, vSGLT^29^ and Mhp1^28^, both of which have 1-to-1 stoichiometries, fail to fully open their outer gates in high Na^+^, while cysteine accessibility studies of LeuT and SERT, which have 2-to-1 stoichiometries, both require Na^+^ binding to open the outer gate^34^. Similarly, double electron–electron resonance studies on LeuT reveal that high Na^+^ levels cause the outer gate to open^35^. Thus, we wondered whether we could engineer in a second Na^+^ site to hSGLT2 by mutating the residue A395 to T (the Na3 binding residue present in hSGLT1). Although hampered by very low expression levels in oocytes, the results partially corroborate the gating hypothesis in that aglycon tails and phlorizin showed lower levels of inhibition to the mutant hSGLT2 than the wild type (Supplementary Table 2).

In summary, we propose a structural model that explains inhibitor binding to the Na^+^-bound, outward-facing conformation of hSGLTs that largely accounts for their functional activity. While we have identified two independent components of subtype selectivity, it still remains to be shown that these elements, together with saturated Na2 occupancy, fully account for the higher potency of the drugs to the renal hSGLT2 isoform. The first component is the formation of a hydrophobic cage surrounding the central ring of the aglycon in hSGLT2, but only partially in hSGLT1. The additional aromatic residue in hSGLT2 is H268 on an extracellular loop that forms a cap over the bound inhibitor. The second component is differences in sodium binding, where the lower potency of inhibitor binding to hSGLT1 is due to the second sodium stabilizing the transporter in an open conformation that lacks stabilizing interactions between the inhibitor and elements of the transporter found on the mobile extracellular gate. Further validation of our predictions awaits the crystal structures of human SGLTs, molecular simulations, and functional assay on mutants; together, these studies will lead to improved drugs.

## Methods

### Sequence alignment and homology modeling

Both a primary sequence alignment (EMBOSS stretcher^15^) and a structural alignment (MatchMaker^16^ within Chimera v1.10.1^36^) were determined for vSGLT (PDB ID 3DH4) and SiaT (PDB ID 5NV9 throughout) followed by hand adjustments to arrive at a consensus alignment used for model building. hSGLT1 and hSGLT2 were added to the alignment based on previously published alignments to vSGLT^10^. Outward-facing models were then created with this alignment using Modeller v9.15^17^ and the transmembrane stretches of TM1-10 from SiaT and the extracellular loops from vSGLT (PDB ID 3DH4) as templates. Outward-facing vSGLT models also included TM0 and a C-terminal tag from the apo vSGLT structure (PDB ID 2XQ2). In all cases, one hundred models were generated using the variable target function method^37^, and the best model was selected based on DOPE^38^ score and visual inspection.

### MD simulations

Models were oriented in the membrane using the OPM server^39^ or superposition to previously oriented models followed by embedding with CHARMM-GUI^40^ in a 1-palmitoyl-2-oleoyl-*sn*-glycero-3-phosphocholine membrane. Final systems 112,000 atoms in size were neutralized in 150 mM NaCl. The CHARMM36^41^ parameter set with the TIP3P water model^42^ was used with GROMACS v2016.1^43^ for the MD engine. Equilibration consisted of 10,000 steps of minimization, or until maximum forces dropped below 5000 kJ/mol/nm, followed by 75 ps of NVT (constant-temperature, constant-volume) dynamics and then 0.3 ns of NPT (constant-temperature, constant-pressure) dynamics. During equilibration, restraints on the bound ions, backbone, side chain, lipid heavy atoms, and dihedral angles of the lipid tails were reduced from 2–10 kcal/mol/Å^2^ to zero. Production runs ranged from 7 to 30 ns. Temperature (303.15 K) was maintained using the Berendsen^44^ algorithm during equilibration followed by Nosé–Hoover^45^ for production using a 1 ps coupling time constant. Pressure (1 bar) was maintained with a Berendsen^44^ barostat followed by a Parrinello–Rahman^46^ barostat with a 5 ps period. The time step was 1 fs during equilibration and 2 fs during production. Hydrogen atoms were constrained with LINCS^47^, long-range electrostatics were computed with particle mesh Ewald, and van der Waals and Coulombic interactions were switched between 10 and 12 Å.

The best hSGLT1–phlorizin model from the docking run was simulated with Na^+^ bound to the Na2 and Na3 sites. The system was again prepared with the CHARMM-GUI webserver in an identical manner to the Na^+^ simulations and converted with in-house scripts into the AMBER format. Simulations were carried out using the ff14SB AMBER^48^ parameter set for the protein, the Joung–Cheatham^49^ parameters for the monovalent ions, LIPID17^50^ for the lipids, and the gaff^51^ force field for phlorizin. The TIP3P model was used to simulate the water. Phlorizin geometry was first optimized and its electrostatic potential was calculated with Gaussian v9-E01^52^ with the 6–31 G* basis set. Next, the RESP^53^ charges were fitted into the electrostatic potential with antechamber^54^ and the force field parameters, topology, and starting coordinates were generated with AmberTools. Simulations were carried out with the pmemd^55^ MD engine on GPUs. Minimization consisted of 10,000 steps, switching from the steepest descent algorithm to conjugated gradient after 5000 steps. The system was then gradually heated from 0 to 303.15 K over 15 ps, and harmonic restraints with a spring constant of 10 kcal/mol/Å^2^ were applied to all heavy atoms except water oxygens. After reaching 100 K, we switched from NVT to NPT. After 105 ps, force constants of 10, 5, and 2.5 kcal/mol/Å^2^ were applied to the substrates and nearby residues (within 5 Å), protein, and lipid headgroups, respectively. Restraints were gently removed over the next ~8 ns, and the system was simulated for 1 μs. Pressure (1 bar) was maintained using a semi-isotropic pressure tensor and the Berendsen barostat. Temperature was maintained with the Langevin thermostat with a friction coefficient of 1 ps^−1^. The SHAKE^56^ algorithm was used with a 2-fs time step. A non-bonded cutoff of 10 Å was used and electrostatics were calculated using the particle mesh Ewald method.

### Virtual screening

The Small Molecule Drug Discovery Suite 2016-1 (Schrödinger LLC)^57^ was used for all the docking calculations. Protein isoforms were prepared in a ready-to-dock-format with the Protein Preparation Wizard. The hydroxyl group orientations and protonation states were assigned at pH 7 using PROPKA^58^ in Epik v3.5. Small molecules were drawn with Maestro v10.5, and their protonation states and tautomers assigned at pH 7.0 ± 2 in Epik v3.5. Conformers were assigned using LigPrep v3.7 with the OPLS force field v3^59^. Docking was performed as follows. Side chain rotamers in the sugar binding site in both hSGLT1 and hSGLT2 were optimized with induced-fit docking^60^. The models were first aligned to the galactose-bound vSGLT structure (PDB ID 3DH4), and restraints were applied to the heavy atoms of the redocked sugar allowing 1.5 Å deviation from the binding mode observed in the X-ray structure. The Standard Precision^61^ scoring function was used for the first docking stage followed by the more accurate XP^62^ function for refinement. Next, small molecules (phlorizin, phloretin, dapagliflozin, dapa-aglycon) were docked into both hSGLT1 and hSGLT2 allowing for small molecule flexibility while keeping the protein rigid to obtain a maximum of 10 optimal binding poses. Prior to docking, a 6 × 6 × 8Å^3^ docking grid was erected on the glucose-optimized binding sites. The *Glide XP* scoring function was employed, and the strain energy was included in the final docking score. Planarity of aromatic groups was enforced and only trans conformations of amide groups were allowed. These 10 poses for each molecule were then rescored with the MM/GBSA protocol to allow side chain relaxation around the small molecule, using the VSGB solvent model^63^ and an implicit membrane. Membrane boundaries were determined based on results from MD simulations, and protein atoms up to 4 Å from the molecule were allowed to move. Final binding poses were picked based on a combination of visual inspection, chemical intuition, and score.

### Chemistry

Dapa-aglycon^22^ was synthesized by copper-catalyzed hydroxylation of the corresponding aryl iodide^64^. A mixture of 1-chloro-2-(4-ethoxybenzyl)-4-iodobenzene (1 mmol), CuI (0.3 mmol), 1,10-phenanthroline (0.6 mmol), and CsOH (3.3 mmol) in dimethyl sulfoxide/water (3:1, 4 ml) was stirred at room temperature for 1 h and then heated to 100 °C for 2 h. After cooling, liquid was transferred to a separatory funnel and diluted with ethyl acetate and washed with 1 M HCl. The organic layer was dried over Na_2_SO_4_ and concentrated under reduced pressure. The crude product was purified by flash column chromatography using hexanes-ethyl acetate as eluents. Proton nuclear magnetic resonance (^1^H NMR) (300 MHz, CDCl_3_) δ 7.21 (1H, d, *J* = 8.5 Hz), 7.09 (2H, d, *J* = 8.4 Hz), 6.83 (2H, d, *J* = 8.4 Hz), 6.62 (1H, dd, *J* = 8.5, 2.7 Hz), 6.55 (1H, d, *J* = 2.6 Hz), 4.76 (1H, s), 4.01 (2H, dd, *J* = 13.9, 6.9 Hz), 3.96 (2H, s), 1.40 (3H, dd, *J* = 6.9, 6.9 Hz). The activity of this dapa-aglycon was consistent with that observed previously^22^.

### Molecular biology

Mutants of hSGLT1 in pBluescript^65^ and hSGLT2 in pT7TS^66, 67^ were prepared using the QuickChange site-directed mutagenesis kit (Statagene La Jolla, CA, USA), using the primers provided in Supplementary Table 3. Capped wild-type and mutant cRNA was synthesized from linearized plasmids using T3 and T7 mMessage mMachine kits (Ambion).

### Functional expression of hSGLT1 in oocytes

*Xenopus laevis* (Nasco) oocytes (V–VI) were prepared as previously described^20^ and injected with 50 nl of cRNA (1 μg/μl). In the case of hSGLT2, co-injection of MAP17 cRNA was required for transport activity^67, 68^. hSGLT1 and hSGLT2 activity was confirmed by the measurement of sodium-dependent uptake of 50 μM α-methyl-d-glucopyranoside as described previously^20^. Briefly, oocytes were incubated for 30 min in the presence of 50 μM αMDG (5 μM [^14^C]αMDG) in a buffer containing (in mM) 100 NaCl, 2 KCl, 1 MgCl_2_, 1 CaCl_2_, and 10 *N*-(2-hydroxyethyl)piperazine-*N*′-(2-ethanesulfonic acid) (HEPES)/Tris (pH 7.5). After incubation, oocytes were rinsed thoroughly with ice-cold buffer containing (mM) 100 choline Cl^–^, 2 KCl, 1 MgCl_2_, 1 CaCl_2_, and 10 HEPES/Tris (pH 7.5), individually lysed with 5% sodium dodecyl sulfate, and assayed for radioactivity.

### Electrophysiology

Injected oocytes were incubated at 18 °C, and after 3–4 days, two electrode voltage clamp experiments were performed as previously described^20^. Briefly, SGLT inward glucose currents were measured as a function of the external Na^+^ (0–100 mM NaCl), sugar (0–100 mM αMDG), and inhibitors (0.001–100 μM). Steady-state and hSGLT1 capacitive currents were recorded using voltage steps from a holding voltage, *V*_h_ of −60 mV, to voltages in the range of −180 to +50 mV in 20 mV increments. The apparent affinity of αMDG, *K*_0.5_, was estimated from plots of the inward current as a function of the external αMDG concentration. Under our experimental conditions, *K*_0.5_ is a close approximation of the αMDG binding constant to the Na^+^-bound outward conformation of SGLT. The apparent affinity for Na^+^, *K*_0.5_, was estimated from plots of the αMDG inward current as a function of external Na^+^ concentration. For hSGLT1 the interpretation of *K*_0.5_ is more complex owing to the presence of two Na^+^ binding sites. In this case, the *K*_0.5_ is a lumped coefficient for Na^+^ binding to the two sites in the outward-facing hSGLT1 conformation. The inhibitor constants for phlorizin and derivatives were determined by: (i) measuring the effect of increasing concentrations of inhibitor on the αMDG inward currents measured in presence of NaCl (100 mM) and a αMDG equivalent to *K*_0.5_ of the specific mutant or wild type^22^, and (ii) from the inhibitor effect on hSGLT1 capacitive currents in the absence of sugar^20^. The Na^+^-to-αMDG stoichiometry was determined from the reversal potential measurements using the Gibbs free energy as previously described^67^. The stoichiometry determinations were reinforced by estimates of αMDG and Na^+^ Hill coefficients, and in some cases, by measurement of ^22^Na and [^14^C]αMDG uptakes under voltage clamp^20, 21^.

### Na^+^ binding site analysis

We compiled a non-redundant protein structural database via the Advanced Search dialog at rcsb.org with the following search criteria (3 June 2018): {Chemical ID(s): NA and; Polymeric type is Free and; Experimental Method is X-RAY and; Resolution is between 0.0 and 2.5 and; Sequence Length is between 40 and 100000 and; Chain Type: there is a Protein chain but not any DNA or RNA and; XrayRefinementQuery: refine.ls_R_factor_obs.comparator = between refine.ls_R_factor_obs.min = 0 refine.ls_R_factor_obs.max = .3 and; Representative Structures at 50% Sequence Identity}. The resulting database was comprised of 1750 proteins containing sodium ions, filtered at 50% sequence similarity and with X-ray resolution ≤2.5 Å and *R*_obs_ ≤0.3. The PDB files were downloaded from the RCSB website, and biological assemblies were created via the python program Prody^69^. Labels of unique chains (by sequence similarity) were saved via a custom table of the RCSB search results. The protein structures were protonated by the program Reduce^70^ with N/Q/H residues allowed to flip.

## Electronic supplementary material

Supplementary Information

Supplementary Movie 1

Description of Additional Supplementary Files

## Data Availability

Data supporting the findings of this manuscript are available from the corresponding authors upon reasonable request. A reporting summary for this Article is available as a Supplementary Information file.
