# Supplementary material for: Inhibitor binding mode and allosteric regulation of Na+-glucose symporters
Source: Nat Commun. 2018 Dec 7;9:5245. doi: 10.1038/s41467-018-07700-1 (PMC6286348; doi:10.1038/s41467-018-07700-1)
Supplement: Supplementary file 1 — Supplementary Information [file 41467_2018_7700_MOESM1_ESM.pdf]

Inhibitor binding mode and allosteric regulation of Na^+-glucose symporters

Paola Bisignano, Chiara Ghezzi, Hyunil Jo, Nicholas F. Polizzi, Thorsten Althoff, Chakrapani Kalyanaraman, Rosmarie Friemann, Matthew P. Jacobson, Ernest M. Wright, and Michael Grabe

Supplementary Information

This file includes:

Supplementary Methods

Supplementary Figures 1 to 7

Supplementary Movie 1

Supplementary Tables 1 to 3

Supplementary References

Supplementary Methods

Docking to an inward-facing hSGLT1 model

We generated one hundred inward-facing models of hSGLT1 with Modeller v9.15 using vSGLT (PDB ID 3DH4) as a template and the alignment in Supplementary Fig. 2. The best model was chosen based on a combination of the DOPE score (#1 of 100) and visual inspection of the binding site rotamers. We then docked glucose into the hSGLT1 model following the IFD protocol described in the Methods of the main text. Our hSGLT1-glucose model not only recapitulates the hydrogen-bond network (H83/Q69, E102/E88, W291/W264, K321/K294, and Q457/Q428 – hSGLT1/vSGLT numbering) and stacking interactions (Y290/Y263 – hSGLT1/vSGLT numbering) observed in 3DH4, (Supplementary Fig. 1a) but also agrees with mutagenesis data[1]. Next, we attempted to dock phlorizin into this model. In all top 10 poses, the sugar moiety failed to bind in the sugar binding pocket, most likely due to steric clashes

between the aglycon tail and inner gate residues Y290 and N78. The #1 ranked pose occupies the intracellular vestibule connected to the cytoplasm, where prominent interactions are formed with Y153 and K157 on TM3, Y290 and D294 on TM6 and N78 and H83 on TM1 (Supplementary Fig. 1b). Although some interactions in the sugar site are maintained (with residues H83 and Y290), the model lacks most of the other polar interaction with the sugar moiety. Moreover, residues in TM3 have not been implicated in binding at all[1] suggesting that the inward-facing model is not suitable for phlorizin docking.

Sequence alignment

We employed two independent alignment procedures to assess the accuracy of our final alignment: a sequence-based alignment[2] and a structural alignment[3] (Supplementary Fig. 2). The structural alignment was derived from a superposition of vSGLT (PDB ID 3DH4, chain A) and SiaT (PDB ID 5NV9). Since structure is often more conserved than sequence, the structural alignment provides a more robust comparison between the two proteins; however, it can fail for proteins that adopt multiple structural states, which is the case for transporters that move between inward- and outward-facing conformations. The two alignment procedures generally agree throughout the TM regions (~70% agreement). For the 10 core helices that make up the LeuT fold, the methods agree exactly in 5 of the 10 helices (TMs 1, 2, 3, 7, and 10). There is partially agreement, in either the N or C terminus, for the remaining 5 core-TM helices, discussed below. Specifically, within the substrate bundle, where transported molecules and inhibitors are known to bind, both methods produced the same alignment for TM1, 2, and 7. The final segment that makes up the substrate bundle (TM6) is agreed upon in the N-terminus, but not the C-terminal side.

Next, we resolved differences between both methods. The easiest segments to resolve were TM5, 6, and 8 where there was good agreement throughout much of the sequence. Both alignments agree on the N-terminal side of the helices; however, the sequence alignment does not create a gap for TM6 and 8, which motivated our choice of this alignment. The structural alignment was chosen for TM5 for the same reason. The alignments of TM3, 4, and 9 were more difficult to address due to low sequence identity and/or less agreement between the two alignment methodologies. For TM3, both methods agree despite an incredibly low sequence identity for this segment. Unfortunately, both methods introduce a gap in the middle of the helix so that V141 in vSGLT fails to align to a residue in SiaT. We chose to close the gap up by retaining the alignment on the N-terminal side of the helix, which has a pattern of basic residues that are conserved in both transporters. TM4 is a short segment, and both alignment methods disagree throughout the entire stretch. Examining the structural alignment, it is clear that there is a major shift in the position of TM4 relative to the rest of the segments during transport. For this reason, we decided to adopt the result suggested by the sequence alignment. Finally, for TM9 there is a large discrepancy between the alignments produced between both methods, and while we would typically lean toward the structural alignment, TM9 undergoes a large motion with respect to the rest of the protein during the transport process. For this reason, we believe that the structural alignment is quite poor, and we adopted the result from the sequence alignment, which has a much higher identity in this region (~28%) compared to the structural alignment.

The sugar binding mode of vSGLT is conserved during transport

The hydrogen bonding pattern between the sugar bundle residues (Q69, E88, S91, N260, and K294) and the polar groups on the sugar is conserved between the inward-facing X-ray structure[4] and our outward-facing model. However, there is one noticeable exception – Q428 on the outer end of TM10 is too far away to contact the sugar in the outward-facing state because the distal end of the helix has moved to open up the outer gate. Distance based spectroscopic experiments show that the presence of galactose favors an outward-occluded state, in which TM9-10 closes over the galactose[5], which would fully coordinate the molecule. Our model is consistent with this result, since the sugar is missing interactions on the C6 hydroxyl group in the outward-open state, and it is likely that TM9-10 closure would produce very little, if any, steric clashes (Supplementary Fig. 4c). Regardless, the high level of agreement between the binding mode of galactose in this outward-facing homology model and the inward-facing X-ray structure provides additional confidence in our alignment between SiaT and vSGLT, and our ability to make faithful homology models.

Sodium site stability in hSGLT1

We setup four short molecular dynamics simulations of hSGLT1 in which the Na^+ was present at either the Na1 or Na3 site in single or double occupancy with another Na^+ in the conserved Na2 site (Supplementary Table 1). Both attempts to simulate Na^+ at the Na1 position failed to provide a stable configuration (Supplementary Fig. 4). In the absence of a Na2 ion, the Na1 ion quickly departs from the initial site placement and moves toward the Na2 site over the 10 ns simulation (Supplementary Fig. 4a). In the presence of a Na2 ion, the Na1 site is even more unstable and the ion leaves the protein to the extracellular space in 3 ns (Supplementary Fig. 4b).

Counter to this initial finding, Na^+ in the Na3 site is stable in the presence or absence of the Na2 ion when simulated 10 ns or 30 ns, respectively (Supplementary Fig. 4c,d).

Na^+ binding site analysis

We catalogued Na^+ binding sites in unique chains of the structural database using the program Probe[6,7]. For an input protein structure, Probe outputs a list of all residues within van der Waals contact (or closer) to a Na^+ ion. Residues with contact type "wc" (wide contact) from Probe were not included in Na^+ coordination spheres, which excludes the more weakly coordinated residues from consideration. Supplementary Fig. 6a, b, and c were compiled from the binding site data.

The sodium-coupled sialic acid symporter (PDB ID 5NV9, resolution of 1.95 Å) and sodium-calcium exchanger proteins (PDB ID 5HYA, resolution of 1.90 Å) shown in Supplementary Fig. 5b,c were discovered to have similar binding sites to hSLGT1 (panel a) after searching through Na^+ binding sites that featured Ser/Thr side chain residues at relative positions i and i + 1 in sequence. Similar binding sites to 5NV9 shown in Supplementary Fig. 5d were discovered via searching through Na^+ binding sites that featured a bidentate Glu or Asp residue (green) as observed in the homology models hSLGT1/2 and the 5NV9 crystal structure. Structural similarity between 5NV9 and other binding sites was quantified via superposition and root mean squared deviation (RMSD) between the following atoms. The four carboxylate atoms of D182 (CB, CG, OD1, OD2) from 5NV9 were mapped to those in the bidentate Asp/Glu of the query binding site (CB/CG, CG/CD, OD1/OE1, OD2/OE2). The Na^+ ions of each site were also included in the superposition. The three

remaining oxygen atoms of the 5NV9 site (O_{342}, OG_{345}, OG_{346}) were mapped sequentially onto all possible permutations of three oxygen atoms in the query binding site; the binding sites were then superimposed and the RMSD was calculated. The superposition with the lowest RMSD was returned.

Supplementary Figures

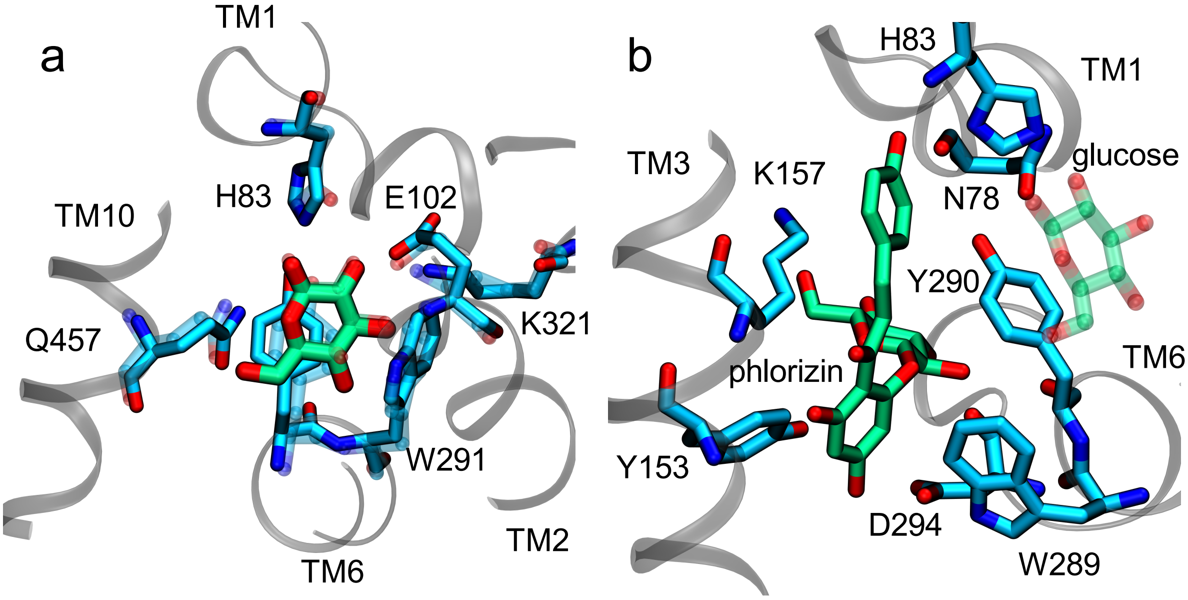

Supplementary Figure 1. Sugar/phlorizin docked into inward-facing model of hSGLT1.

(a) Glucose (green/red molecule) binding to an inward-facing hSGLT1 model recapitulates the binding mode of galactose (transparent green/red molecule) observed in the vSGLT co-crystal[4]. The hSGLT1 binding site residues (cyan/red/blue) align well with the residues from vSGLT (transparent). Numbering corresponds to hSGLT1. **(b)** Phlorizin (green/red molecule) docked into the inward-facing model of hSGLT1. All phlorizin docked poses fail to occupy the sugar binding site (indicated by the transparent glucose molecule), as demonstrated by the highest ranked pose shown here. The molecule is poorly packed, and it fails to make good π-π interactions with the surrounding aromatic residues.

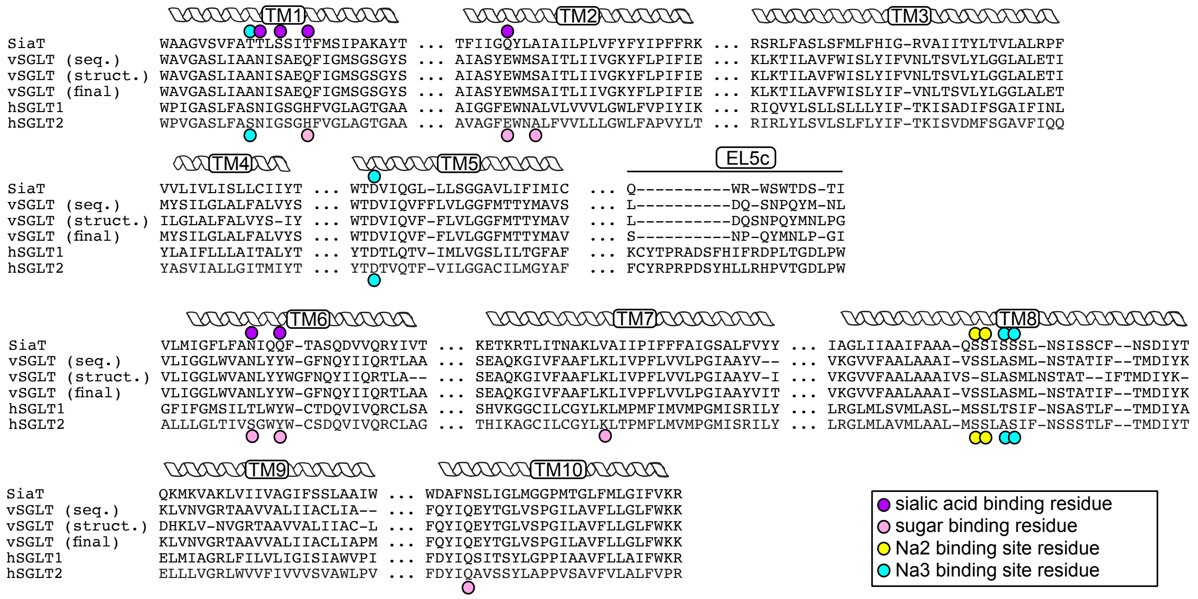

Supplementary Figure 2. Sequence alignments of SGLTs to SiaT.

vSGLT was aligned to SiaT using a sequence-based alignment or a structure-based alignment method indicated by seq. and struct., respectively. Inclusion of hSGLT1 and hSGLT2 in the alignment follows from previously published alignments to vSGLT. The sequence used for all homology modeling is indicated by final and is described in the Supplementary Methods. Key ion and substrate binding residues in each molecule are identified in the legend based on a number of structural and functional studies carried out on each transporter. Only TM domains are shown except for ELC_5.

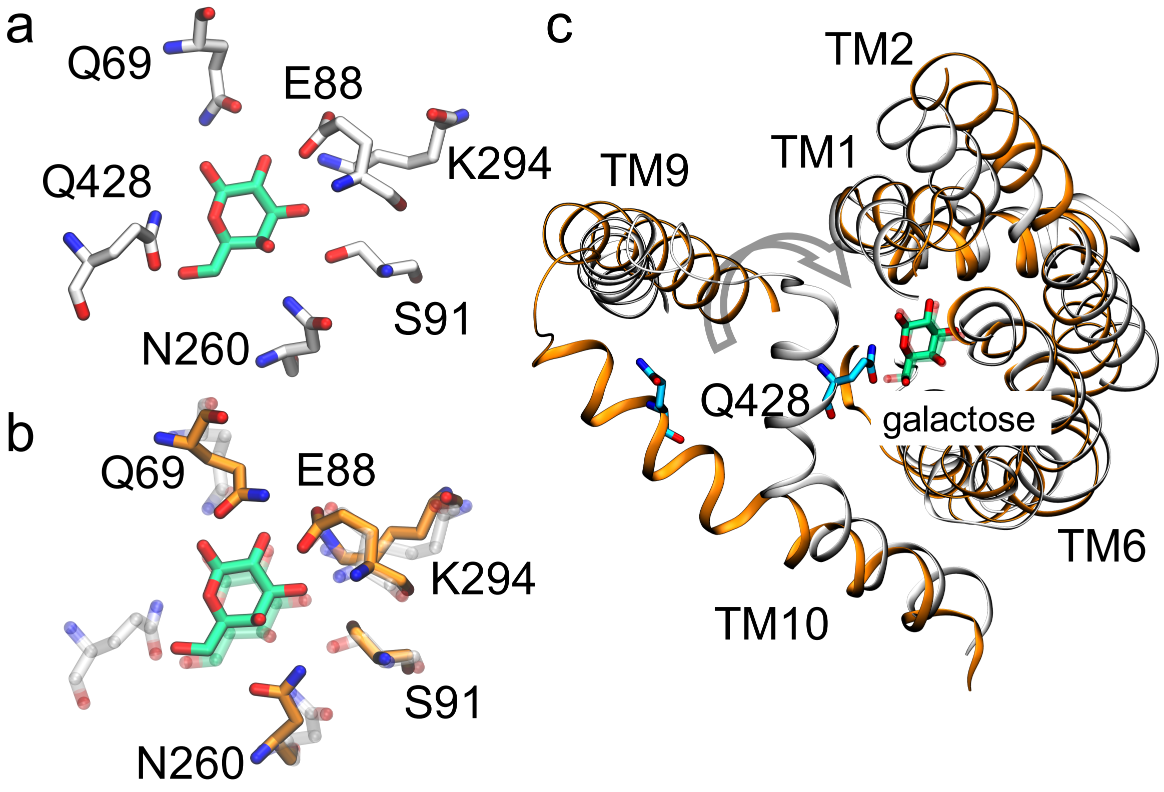

Supplementary Figure 3. Comparison of galactose-bound outward/inward vSGLT states.

(a) Galactose (green/red sticks) engages its polar groups with the sugar bundle residues Q69, E88, S91, N260, K294, and Q428 on TM10 in the inward-facing vSGLT X-ray structure (PDB ID 3DH4). **(b)** Galactose (solid green/red) docked into the outward-facing model perfectly reproduces (RMSD < 0.9 Å) the X-ray pose (transparent green/red) of the inward-facing state. The configuration of all binding site residues, except Q428 on TM10, are preserved. **(c)** The superposition of the outward-facing model (orange from b) onto the inward-facing structure (white from a) reveals that Q428 interacts with the sugar only upon outer gate closure (inward-facing conformation). In all panels, nitrogen atoms are blue, oxygen red, and carbon are white in the inward-facing structure and orange in the outward-facing model.

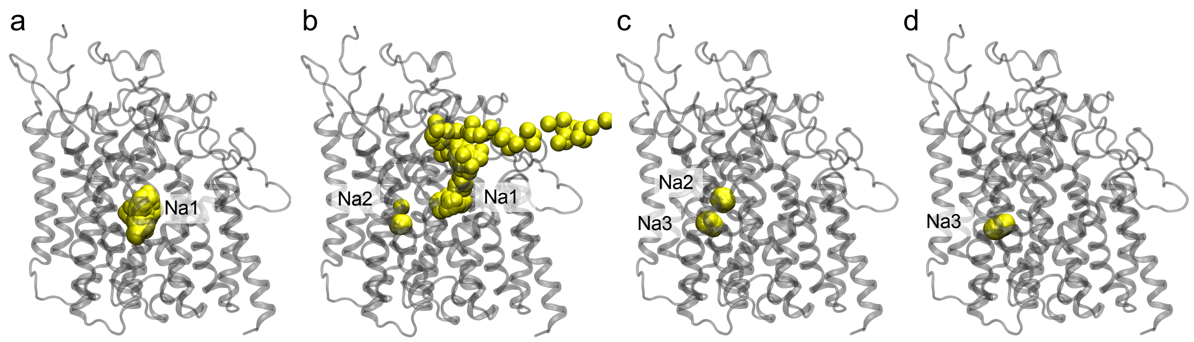

Supplementary Figure 4. Ion stability at Na sites from MD simulations.

Four hSGLT1 simulations were performed to assess the stability of Na^+ at each putative ion site. Na^+ at Na1 is not stable in single occupancy **(a)** or double occupancy **(b)** with another ion in Na2. The Na1 ion escapes the protein after 3 ns in the latter case. Na^+ in the Na3 site is stable in double **(c)** and single occupancy **(d)** for 10 ns and 30 ns, respectively. In all panels, hSGLT1 is white and bound ions are represented as yellow spheres drawn every 10 ps throughout the trajectory.

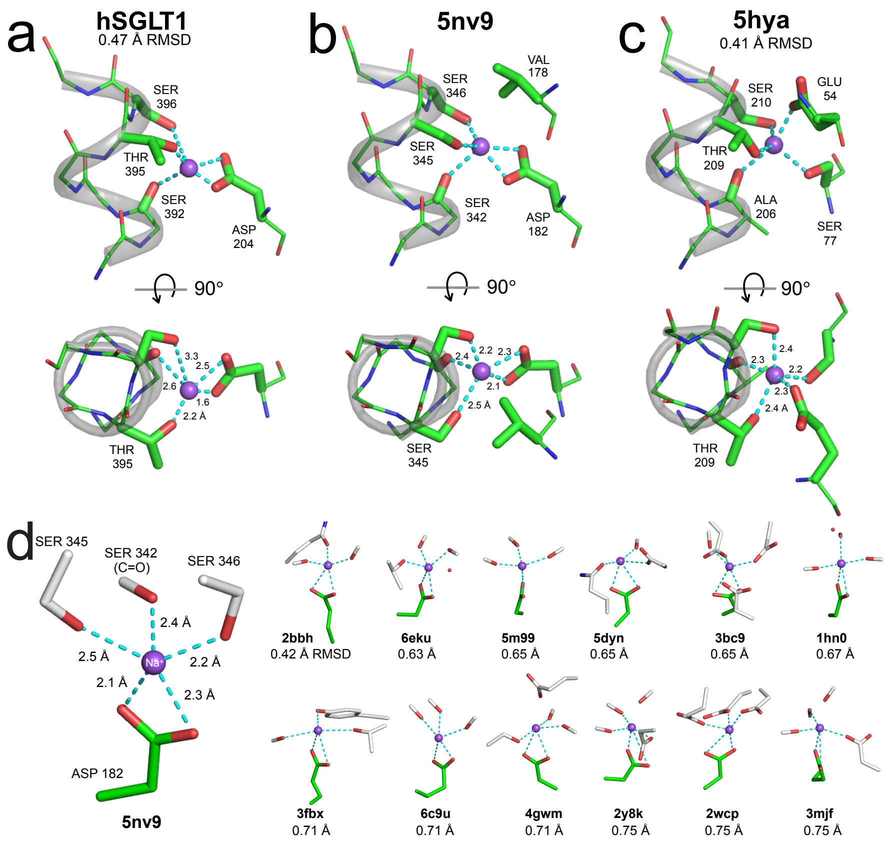

Supplementary Figure 5. The Na3 site of hSGLT1 is similar to many Na$^+$ binding sites.

(a) hSGLT1 has a nearly identical coordination sphere as the Na$^+$-coupled sialic acid symporter (**b**, PDB ID 5NV9), with Ser 345 substituted by Thr. **(c)** The sodium-calcium exchanger (PDB ID 5HYA) contains a structurally similar Na$^+$ coordination motif as hSGLT1 and 5NV9, with root mean squared deviation (RMSD) of 0.41 Å. Coordination between Na$^+$ and oxygen atoms are shown with cyan dashed lines, with labeled distances. Each Na$^+$ coordination sphere in **a**, **b**, and **c** contains five oxygen atoms. Four of these five oxygen atoms (O_{342}/O_{206}, $OG_{345}/OG1_{209}$, OG_{346}/OG_{210}, $OD1_{183}/OG_{77}$) and the Na$^+$ from 5NV9 and 5HYA were superimposed, and the RMSD was calculated with respect to these atoms. The poses shown are those resulting after superposition. Sodium is shown as purple spheres. **(d)** The protein data bank contains many examples of Na$^+$-coordination spheres similar to the Na3 site of hSGLT1. Structural matches to the coordination geometry of 5NV9 (a nearly identical coordination sphere to hSGLT1) are shown with PDB accession codes and RMSD. Dashed, cyan lines highlight the coordination between Na$^+$ and oxygen atoms of the matches that correspond to oxygen atoms in 5NV9. The binding sites from 5NV9 and each structural match were superimposed via carboxylate, Na$^+$, and oxygen atoms (see Methods in the main text); and the RMSD was calculated with respect to these atoms. The poses shown are those resulting after superposition. Bidentate Glu or Asp residues are colored green. Waters are shown as red spheres. Sodium is shown as purple spheres.

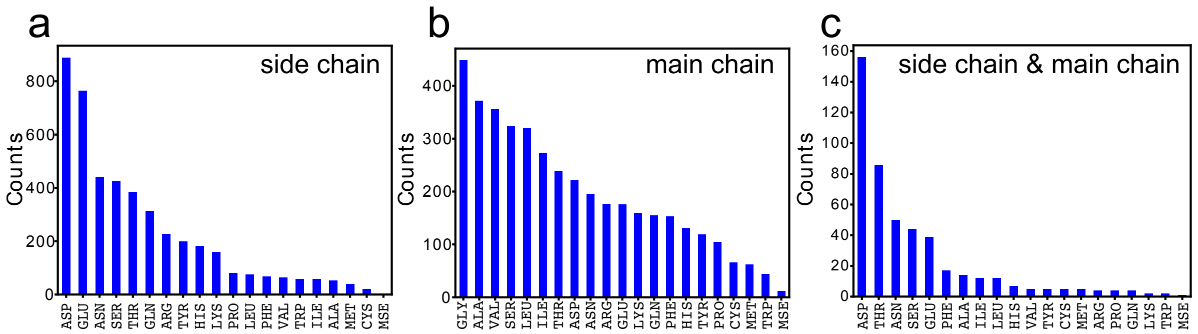

Supplementary Figure 6. Abundancy of Na^+ coordination by residue type.

(a) Number of amino acids in a non-redundant protein structural database that coordinate Na^+ via side chain only. **(b)** Number of amino acids in a non-redundant protein structural database that coordinate Na^+ via main chain carbonyl only. These residues do not coordinate via any side chain atoms. **(c)** Number of amino acids in a non-redundant protein structural database that coordinate Na^+ via both side chain and main chain carbonyl atoms simultaneously.

MSE = selenomethionine.

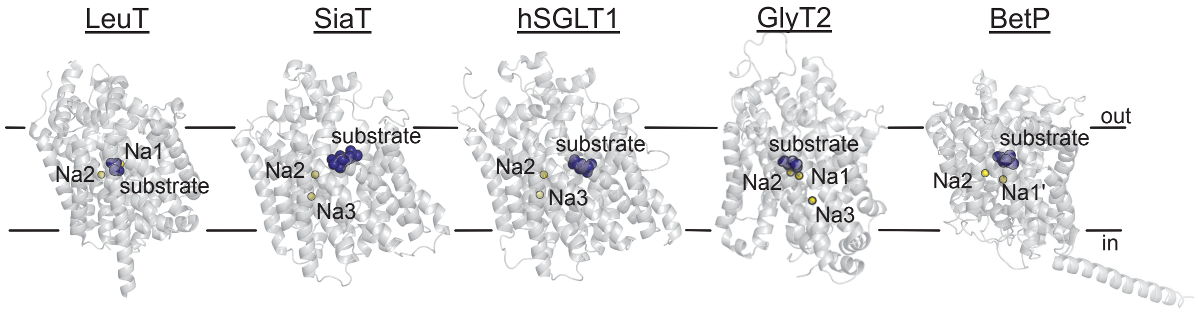

Supplementary Figure 7. Survey of LeuT family members with multiple Na^+ sites.

Structures of five family members that have stoichiometries greater than one. All structures have been oriented in a similar manner with the extracellular side on top. The Na^+ ions are yellow, substrates blue, and approximate membrane boundaries are black lines. LeuT and SiaT are X-ray structures with density observed for both putative Na^+ sites, while hSGLT1 and GlyT2 are homology models. BetP is an X-ray structure, but the Na1' site was determined from computational and functional data. All members have a putative 2-to-1 stoichiometry except GlyT2, which is 3-to-1. The structures of all LeuT family members solved to date contain the Na2 site, and this site engages TM1, TM5 and TM8, but not the substrate. For LeuT the Na1 site directly coordinates the substrate as well as TM1, TM6, and TM7. For BetP, the location of the additional sodium binding site, termed Na1', between TM6 and TM3 was hypothesized based on symmetry arguments coupled with MD simulations, and then validated with functional assays[8]. Unlike Na1, Na1' does not coordinate the substrate. The homology model of the GlyT2 transporter was constructed based on LeuT, and the authors hypothesized that it contains the canonical Na2 site, a Na1-like site that coordinates the substrate, and a third binding site, that they called Na3[9]. The site of Na3 was identified via combination of simulation and functional assay, and it is located far from all other sites near the inward gate coordinating TM3, TM6, and TM10. The 1.9 Å X-ray structure of SiaT revealed two Na^+ ions, and Na^+ Hill coefficient of 1.5 – similar to values reported for hSGLT1, which is well established to have a 2-to-1 stoichiometry. MD simulations revealed that Na^+ in this new site called Na3 – not to be confused with the Na3 site in GlyT2 – is very stable[10]. The Na3 site in SiaT and hSGLT1, based on homology, is below Na2, engaging TM1, TM5, and TM8.

Supplementary Tables

	Setup 1 (10 ns)	Setup 2 (7 ns)	Setup 3 (10 ns)	Setup 4 (30 ns)
Na1	√	√		
Na2		√	√	
Na3			√	√

Supplementary Table 1. Ion occupancies for four distinct simulated systems.

Check marks indicate that Na^+ is present in the structure at the start of the simulation.

Transporter	I_{max} (nA)	$K_{0.5}$ αMDG (mM)	$K_{0.5}$ Na^+ (mM)	Hill coefficient	K_i phlorizin (μM)	K_i phloretin (μM)	K_i dapagliflozin (μm)	K_i dapa-aglycon (μm)
hSGLT1 wild-type	-750±10	0.9±0.1	36±1	1.7±0.03	0.22±0.04	55±12	0.45±0.02	425±50
hSGLT1 T395A	-100±6	34±4	104±50	1.1±0.3	0.35±0.10	20±7	0.4±0.1	187±80
hSGLT2 wild-type	-75±8	4.4±0.1	22±1	1.0±0.1	0.03±0.01	27±3	0.004±0.001	110±30
hSGLT2 A395T	-10±5*	4±0.6*	30±10*	1.3±0.5*	0.042±0.01[#]	129±70[#]	0.002±0.006[#]	>600"

Supplementary Table 2. Na3 site mutant properties.

I_{max} was measured at -50 mV in presence of 10 mM αMDG and 100 mM NaCl, hSGLT1 wild-type, T395A, and hSGLT2 wild-type values are means ± SEM of n ≥ 6 oocytes, and hSGLT2 A395T values are means ± SEM of n = [*]4, [#]3, and "2 oocytes.

Mutant	Primer sequence
T395A	FW: CTCATGAGCTCCCTGGCCTCCATCTTCAAC<br>RV:GTTGAAGATGGAGGCCAGGGAGCTCATGAG
D268H	FW: CCACATCTTCCGACATCCCCTCACGGG<br>RV:CCCGTGAGGGGATGTCGGAAGATGTGG
R267C	FW: CTCCTTCCACATCTTCTGTGATCCCCTCACGGGAG<br>RV: CTCCCGTGAGGGGATCACAGAAGATGTGGAAGGAG
hSGLT2 A395T	FW: GCC GCG CTC ATG TCC TCG CTG ACC TCC ATC TTC AAC AGC AGC<br>RV: GCT GCT GTT GAA GAT GGA GGT CAG CGA GGA CAT GAG CGC GGC

Supplementary Table 3. Primers for the new hSGLT1 and hSGLT2 mutants.

Supplementary References

1 Sala-Rabanal, M. *et al.* Bridging the gap between structure and kinetics of human SGLT1. *Am J Physiol Cell Physiol* **302**, C1293-1305, doi:10.1152/ajpcell.00397.2011 (2012).

2 Myers, E. W. & Miller, W. Optimal alignments in linear space. *Comput Appl Biosci* **4**, 11-17 (1988).

3 Meng, E. C., Pettersen, E. F., Couch, G. S., Huang, C. C. & Ferrin, T. E. Tools for integrated sequence-structure analysis with UCSF Chimera. *BMC Bioinformatics* **7**, 339, doi:10.1186/1471-2105-7-339 (2006).

4 Faham, S. *et al.* The crystal structure of a sodium galactose transporter reveals mechanistic insights into Na+/sugar symport. *Science* **321**, 810-814, doi:10.1126/science.1160406 (2008).

5 Paz, A. *et al.* Conformational transitions of the sodium-dependent sugar transporter, vSGLT. *P Natl Acad Sci USA* **115**, E2742-E2751, doi:10.1073/pnas.1718451115 (2018).

6 Word, J. M. *et al.* Visualizing and quantifying molecular goodness-of-fit: small-probe contact dots with explicit hydrogen atoms. *J Mol Biol* **285**, 1711-1733, doi:10.1006/jmbi.1998.2400 (1999).

7 Word, J. M., Lovell, S. C., Richardson, J. S. & Richardson, D. C. Asparagine and glutamine: Using hydrogen atom contacts in the choice of side-chain amide orientation. *Journal of Molecular Biology* **285**, 1735-1747, doi:DOI 10.1006/jmbi.1998.2401 (1999).

8 Khafizov, K. *et al.* Investigation of the sodium-binding sites in the sodium-coupled betaine transporter BetP. *Proc Natl Acad Sci U S A* **109**, E3035-3044, doi:10.1073/pnas.1209039109 (2012).

9 Subramanian, N. *et al.* Identification of a 3rd Na+ Binding Site of the Glycine Transporter, GlyT2. *PLoS One* **11**, e0157583, doi:10.1371/journal.pone.0157583 (2016).

10 Wahlgren, W. Y. *et al.* Substrate-bound outward-open structure of a Na(+)-coupled sialic acid symporter reveals a new Na(+) site. *Nat Commun* **9**, 1753, doi:10.1038/s41467-018-04045-7 (2018).
