# Supplementary material for: Inhibitor binding mode and allosteric regulation of Na+-glucose symporters
Source: Nat Commun. 2018 Dec 7;9:5245. doi: 10.1038/s41467-018-07700-1 (PMC6286348; doi:10.1038/s41467-018-07700-1)
Supplement: Supplementary file 3 — Description of Additional Supplementary Files [file 41467_2018_7700_MOESM3_ESM.docx]

**Title:** Supplementary Movie 1

**Description:** Simulation of phlorizin bound to hSGLT1. One µs simulation of the hSGLT1-phlorizin complex using all atom MD. The system adopts a partially closed state as the outer gate composed of TM9-10 (ice blue helices) and the EL5 extracellular loop between TM5-6 (red) move over phlorizin (green/red molecule). The simulation reveals that Q457 (cyan/red/blue residue represented as van der Waals) on TM10 comes into contact with the inhibitor. The transporter is represented as ribbons, sodium ions are yellow spheres, phlorizin is represented as van der Waals, and lipids and water were omitted for clarity.
